# Inflammation-based lung adenocarcinoma molecular subtype identification and construction of an inflammation-related signature with bulk and single-cell RNA-seq data

**DOI:** 10.18632/aging.205840

**Published:** 2024-05-20

**Authors:** Yan Gu, Chengyu Bian, Hongchang Wang, Chenghao Fu, Wentao Xue, Wenhao Zhang, Guang Mu, Yang Xia, Ke Wei, Jun Wang

**Affiliations:** 1 Department of Thoracic Surgery, Jiangsu Province Hospital and the First Affiliated Hospital of Nanjing Medical University, Nanjing 210029, Jiangsu, China; 2 Department of Thoracic Surgery, The First People’s Hospital of Changzhou and The Third Affiliated Hospital of Soochow University, Changzhou 213004, Jiangsu, China

**Keywords:** inflammation, immune, lung adenocarcinoma, molecular subtypes, tumor microenvironment

## Abstract

The role of inflammation is increasingly understood to have a central influence on therapeutic outcomes and prognosis in lung adenocarcinoma (LUAD). However, the detailed molecular divisions involved in inflammatory responses are yet to be fully elucidated. Our study identified two main inflammation-oriented LUAD grades: the inflammation-low (INF-low) and the inflammation-high (INF-high) subtypes. Both presented with unique clinicopathological features, implications for prognosis, and distinctive tumor microenvironment profiles. Broadly, the INF-low grade, marked by its dominant immunosuppressive tumor microenvironment, was accompanied by less favorable prognostic outcomes and a heightened prevalence of oncogenic mutations. In contrast, the INF-high grade exhibited more optimistic clinical trajectories, underscored by its immune-active environment. In addition, our efforts led to the conceptualization and empirical validation of an inflammation-centric predictive model with considerable predictive potency. Our study paves the way for a refined inflammation-centric LUAD classification and fosters a deeper understanding of tumor microenvironment intricacies.

## INTRODUCTION

Globally, lung cancer remains the predominant contributor to cancer-induced fatalities, representing 18.0% of such deaths [1–3]. Among the various subtypes, lung adenocarcinoma (LUAD) emerges conspicuously. The formidable characteristics of LUAD, notably its invasive nature and pronounced variability, make the prognosis challenging [4, 5]. The intricacies of the tumor microenvironment (TME) and pervasive mutations diminish the effectiveness of certain interventions, such as targeted therapy and immunotherapy in the context of LUAD [6, 7]. Consequently, identifying and utilizing precise biomarkers to refine LUAD diagnosis is imperative to enable individualized treatment approaches.

Inflammation is associated with myriad ailments, including LUAD [8–11]. In some instances, transient inflammation may bolster anticancer immune activity, whereas in others, chronic inflammatory responses precipitated by therapeutic interventions may fortify tumor resilience [12–14].

The advent of immunotherapy has transformed oncological care, reigniting interest in tumor immunology. However, despite the exploitation of immune checkpoint inhibitors as a therapeutic arsenal, the outcomes for advanced-stage LUAD continue to be suboptimal [15, 16], prompting the need to unearth the underpinnings of immune evasion and develop innovative immunotherapeutic avenues tailored for LUAD.

In this context, our study marks a pioneering effort to shed light on the contribution of inflammation to LUAD initiation, evolution, and therapeutic responsiveness. We identified two inflammation-centric subtypes of LUAD and investigated the disparities between these subgroups concerning their inflammatory signatures, clinicopathological attributes, TME nuances, and responses to immunotherapy. Subsequently, we designed an inflammation-centric gene-predictive model to enhance the overall survival forecasting accuracy of patients with LUAD by employing diverse regression methodologies. Furthermore, we validated the expression trends of inflammation-associated genes by single-cell RNA sequencing (scRNA-seq) data analysis.

## MATERIALS AND METHODS

### Data collection and analysis

The scRNA-seq dataset GSE131907, encompassing 11 tumors and 11 corresponding normal lung samples from LAUD [17], were procured from the GEO (Gene Expression Omnibus) repository. Single cells were selected based on the gene expression observed in at least three cells, with each cell manifesting a minimum of 250 genes. Bulk RNA-seq datasets, comprising survival data, were gathered from repositories such as TCGA-LAUD, GSE3141, GSE37745, and GSE68465. Moreover, the dataset treated with PD-L1 along with its clinical characteristics was retrieved from the GSE78220 database and the IMvigor210 cohort [18].

### Inflammation-related gene characterization, consensus clustering, and principal component analysis

The set of genes related to inflammation was sourced by gene set enrichment analysis (HALLMARK_INFLAMMATORY_RESPONSE). Consensus clustering was using the “ConsensusClusterPlus” package in R. An ideal cluster count between k = 2 and k = 10 was determined, and the process was reiterated 1000 times to obtain robust and consistent results. A cluster map was generated using pheatmap function in the R software. Principal component analysis (PCA) was employed to explore the transcriptional patterns. The analysis was executed using the “limma” package, and results were visualized using the “ggplot2” package in R.

### Hub gene identification

Genes associated with prognosis were identified by univariate Cox regression analysis using the Kaplan–Meier “survival” package (P < 0.05). LASSO Cox regression analysis was performed to minimize gene quantity, followed by multivariate Cox regression analysis using stepwise regression. The predictive accuracy of the risk signature was evaluated by ROC analysis using the “timeROC” package.

### Analysis of immune landscape

The CIBERSORT algorithm was used to analyze the ratios of 22 immune cell subtypes in the TCGA cohort, providing insights into immune cell infiltration [19]. The ESTIMATE algorithm was used to determine the immune and stromal scores, facilitating a deeper exploration of the TME. To confirm variations in immune status among subtypes, both ESTIMATION and ssGSEA were performed [20, 21]. The “cancer-immunity cycle,” comprising seven sequential steps, was analyzed in two subtypes using TIP [22]. These steps included tumor antigen creation (step 1), antigen presentation (step 2), priming and activation (step 3), T cell migration to tumors (step 4), immune cell entry into tumors (step 5), tumor cell recognition by T cells (step 6), and tumor cell apoptosis (step 7). The immunological functions of these steps were assessed in the three subtypes by using TIP.

### scRNA-seq evaluation

TISCH was used to analyze the scRNA-seq data [23, 24]. This single-cell RNA-seq data resource focuses on the TME and offers detailed cell-type annotations at the single-cell level, enabling TME research across various cancers.

### Patient recruitment and tissue collection

Localized NSCLC tumor tissues and corresponding normal tissues (n = 3 each) were procured from patients treated at Jiangsu Province People’s Hospital between 2015 and 2016.

### Western blot analysis

Proteins were isolated from the collected tissues by using radio-immunoprecipitation assay lysis buffer (Thermo Fisher Scientific, MA, USA), supplemented with 1% Halt™ Protease and Phosphatase Inhibitor Cocktail (Thermo Fisher Scientific). Standard western blot procedures were using primary antibodies specific for NF-kB p65 (sc-8008, 1:500) sourced from Santa Cruz Biotechnology (TX, USA) and β-actin (#21338, 1:1000) obtained from Signalway Antibody (MD, USA). For detection, horseradish peroxidase-conjugated secondary antibodies, namely goat anti-rabbit (#L3012, 1:10000) and goat anti-mouse (#L3032, 1:10000) IgG, were used, which were also procured from Signalway Antibody (MD, USA).

### RNA isolation and quantitative real-time PCR (qRT-PCR)

Total RNA was extracted from tissue samples using TRIzol Reagent (Invitrogen, CA, USA). The isolated RNA was then reverse-transcribed to cDNA using a HiScript III 1st Strand cDNA Synthesis Kit (+gDNA wiper) (Vazyme, Nanjing, China). RELA and β-actin primers were procured from RiboBio Company (Guangzhou, China).

### Animal studies

Male BALB/c nude mice, aged six weeks and weighing 15–20 g, were procured from the Model Animal Research Center of Nanjing University (Nanjing, China). The mice were housed in a controlled environment with a 12-hour light/dark cycle and provided access to food and water ad libitum. For the subcutaneous tumorigenesis model, mice were categorized into three groups: RELA Overexpression (RELA-OE), Control, and RELA Short Hairpin RNA (RELA-sh) with seven mice in each group. Stable transgenic LUAD cell lines (RELA-OE, Control, RELA-sh) were prepared as sterile cell suspensions, and 0.1 mL (roughly 5 x 10^6^ cells) of the suspension was injected subcutaneously under the axillary skin of the mice. The mice were finally euthanized via intraperitoneal injection of high-dose pentobarbital (200 mg/kg), and tissues were harvested for subsequent analyses.

### FRAP and imaging

Fluorescence recovery after photo-bleaching (FRAP) was performed using a Stellaris STED confocal microscope with a 63× oil immersion objective. To assess the FRAP in the central region of the protein droplets, the bleaching step was repeated thrice using a 488 nm Argon laser at 60% power. The recovery of fluorescence after bleaching was documented every 2 s for a total duration of 400 s.

### Immunofluorescence

H1299 cells were seeded in 6-well plates. After adhesion, the cells were washed and fixed with 4% paraformaldehyde for 30 min and permeabilized with 0.5% Triton X-100 for 20 min. Blocking was performed with BSA for 1 h. Subsequently, cells were incubated overnight at 4° C with primary antibodies: rabbit anti-RELA (#8242S, Cell Signaling Technology, USA), mouse anti-H3K4me3 (#PTM-160, PTM BIO, USA), and mouse anti-RNA Pol II-S5P (#04-1571, Sigma-Aldrich, USA). Following primary antibody incubation, cells were exposed to fluorescent secondary antibodies (#20000668 and #20000631, 1:3000; Proteintech, Wuhan, China) for 1 h at 25° C, shielded from light. Finally, the cells were stained with DAPI, and images were captured using a Stellaris STED confocal microscope (Leica, Germany).

### Statistical analysis

Statistical analyses were conducted using R software (v4.1.2). Pearson’s or Spearman’s correlation was used for estimating the correlation matrices, and group comparisons were performed using the Wilcoxon test. Survival disparities were evaluated using Kaplan–Meier curves and the log-rank test, with a P-value < 0.05 deemed statistically significant.

### Data availability statement

The authors confirm that the data supporting the findings of this study are available within the article and its Supplementary Materials.

## RESULTS

### Consensus clustering identified two inflammation-based subtypes of LUAD

**Figure 1:**
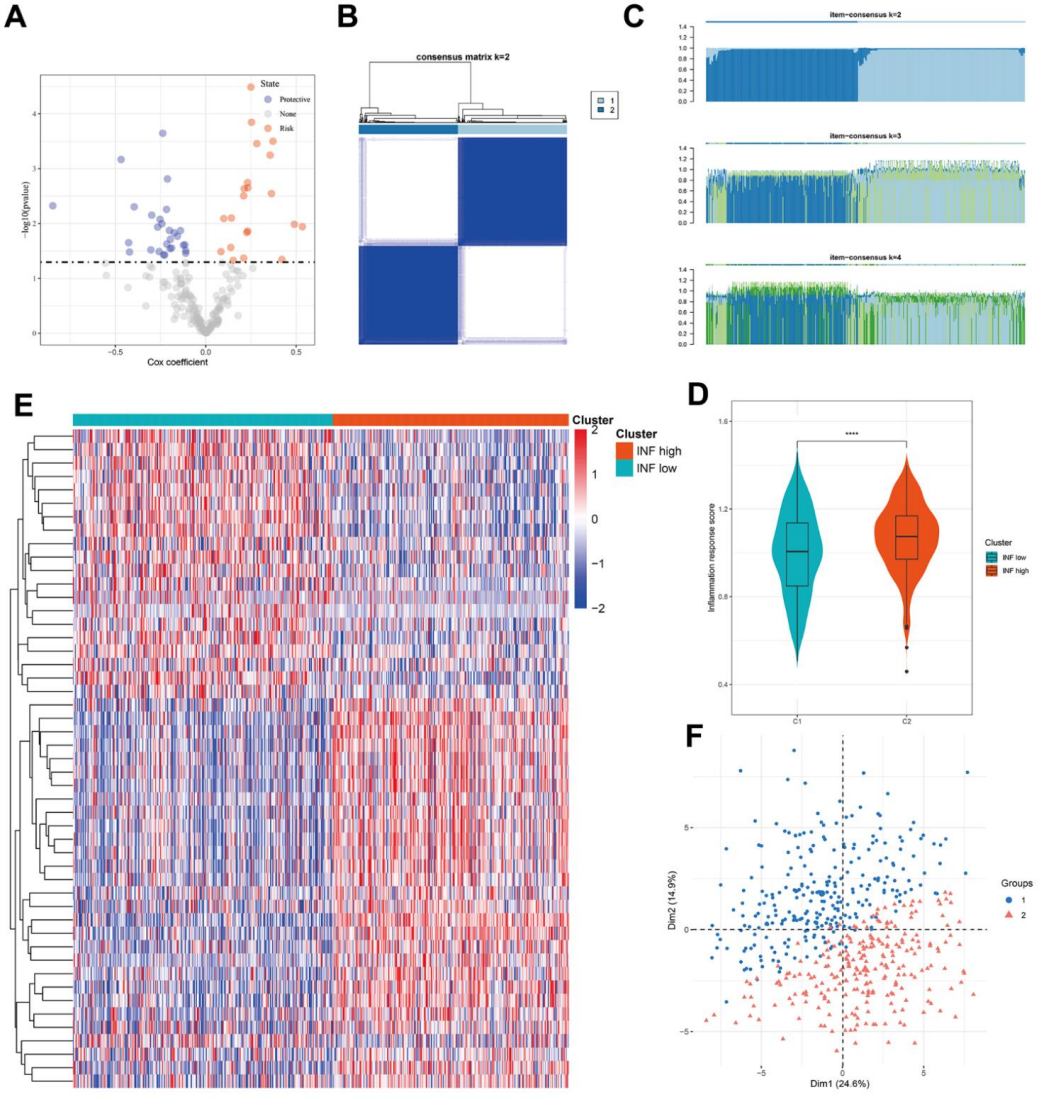
**Identification of two inflammation subtypes in LUAD.** (**A**) Volcano plot of prognosis-related inflammation genes identified by univariate Cox regression analysis. (**B**) Consensus clustering matrix for k = 2. (**C**) Consensus of the items (k = 2–4) in each cluster. (**D**) Violin plots indicating the differences between the 2 subtypes. (**E**) Heatmap of 49 inflammation response gene expression in different subgroups; red represents high and blue represents low expression levels. (**F**) Principal component analysis plots. ****P < 0.0001.

A compendium of 200 inflammation-related genes was assembled, of which 197 were retrieved from TCGA-LUAD cohort. Univariate Cox regression analysis identified 49 genes significantly associated with LUAD prognosis (Figure 1A). Consensus clustering was employed to discern LUAD inflammation-based subtypes, leading to the identification of two distinct clusters within TCGA cohort, with both exhibiting disparate inflammation gene expression patterns (Figure 1B, 1C). The inflammatory response score for each patient was quantified using ssGSEA, which revealed that the C2 cluster had the highest score (Figure 1D). Consequently, C1 was designated as an inflammation-low (INF-low) subtype and C2 as an inflammation-high (INF-high) subtype, with the latter manifesting as elevated inflammatory gene expression (Figure 1E). PCA was performed to compare the transcriptional patterns of these inflammatory subtypes and revealed a clear segregation between the two clusters, indicating distinct transcriptional profiles (Figure 1F).

### Patients stratified into different inflammation subtypes presented distinct prognoses and clinicopathologic features

**Figure 2:**
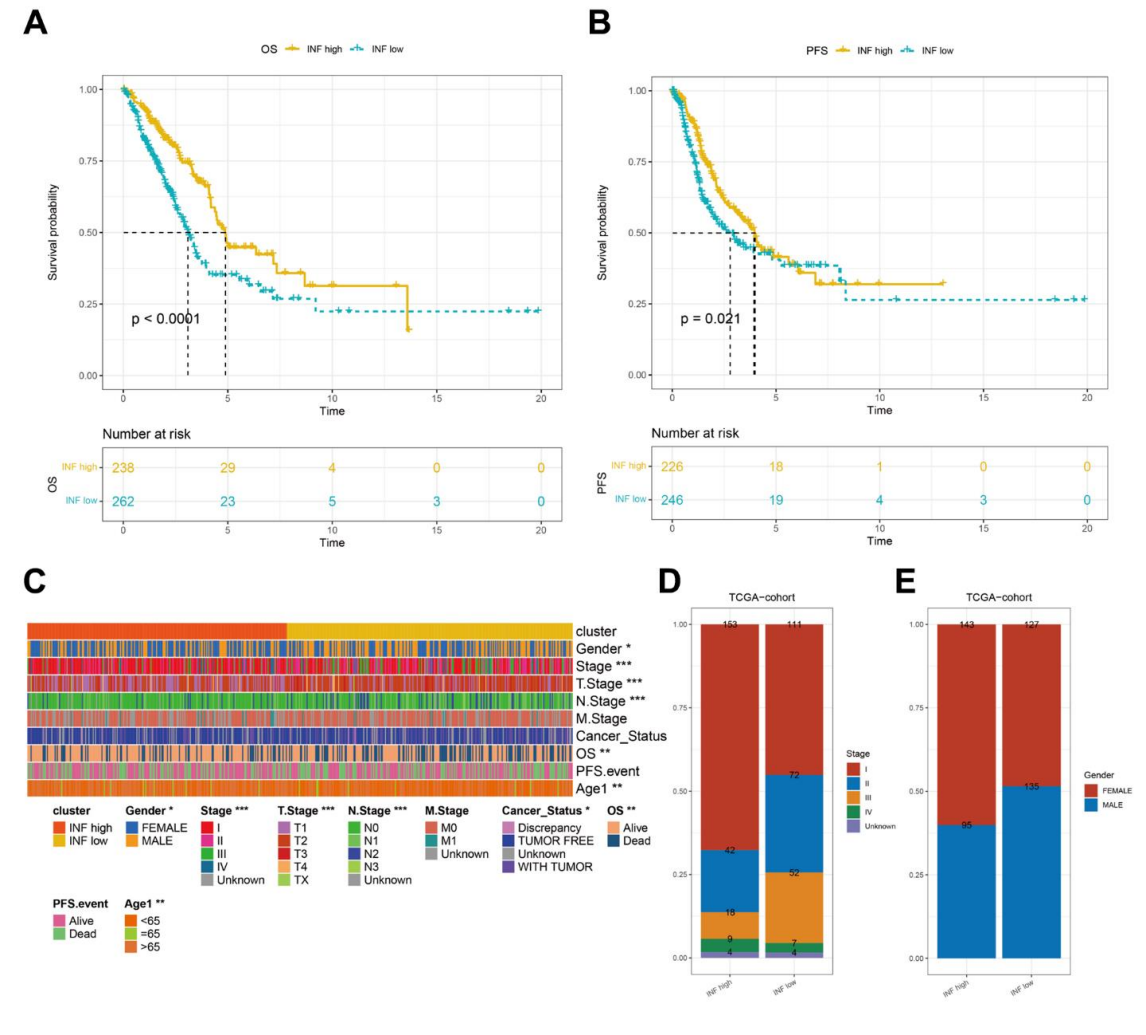
**Differences in the prognostic and clinicopathologic features among the inflammation subtypes.** (**A**, **B**) Kaplan–Meier OS and PFS curves of patients from TCGA cohort assigned as INF-low and -high subtypes. (**C**–**E**) The clinicopathologic features of the subtypes.

Previous studies have established the significant impact of inflammation on the onset and progression of cancer [25]. Survival analyses using TCGA data confirmed that distinct inflammation-based subtypes corresponded to specific clinical outcomes. The INF-low subtype had an unfavorable prognosis, marked by diminished overall survival (OS) and progression-free survival (PFS) (Figure 2A, 2B). A comparative assessment of the clinicopathological characteristics of the two subtypes showed that patients identified with the INF-low subtype were predominantly male and presented with an advanced stage, while experiencing reduced OS duration, being younger, and displaying more adverse cancer effects than those identified with the INF-high subtype (Figure 2C–2E).

### Inflammation-based subtypes were associated with distinct TME features and anticancer immune activities

**Figure 3:**
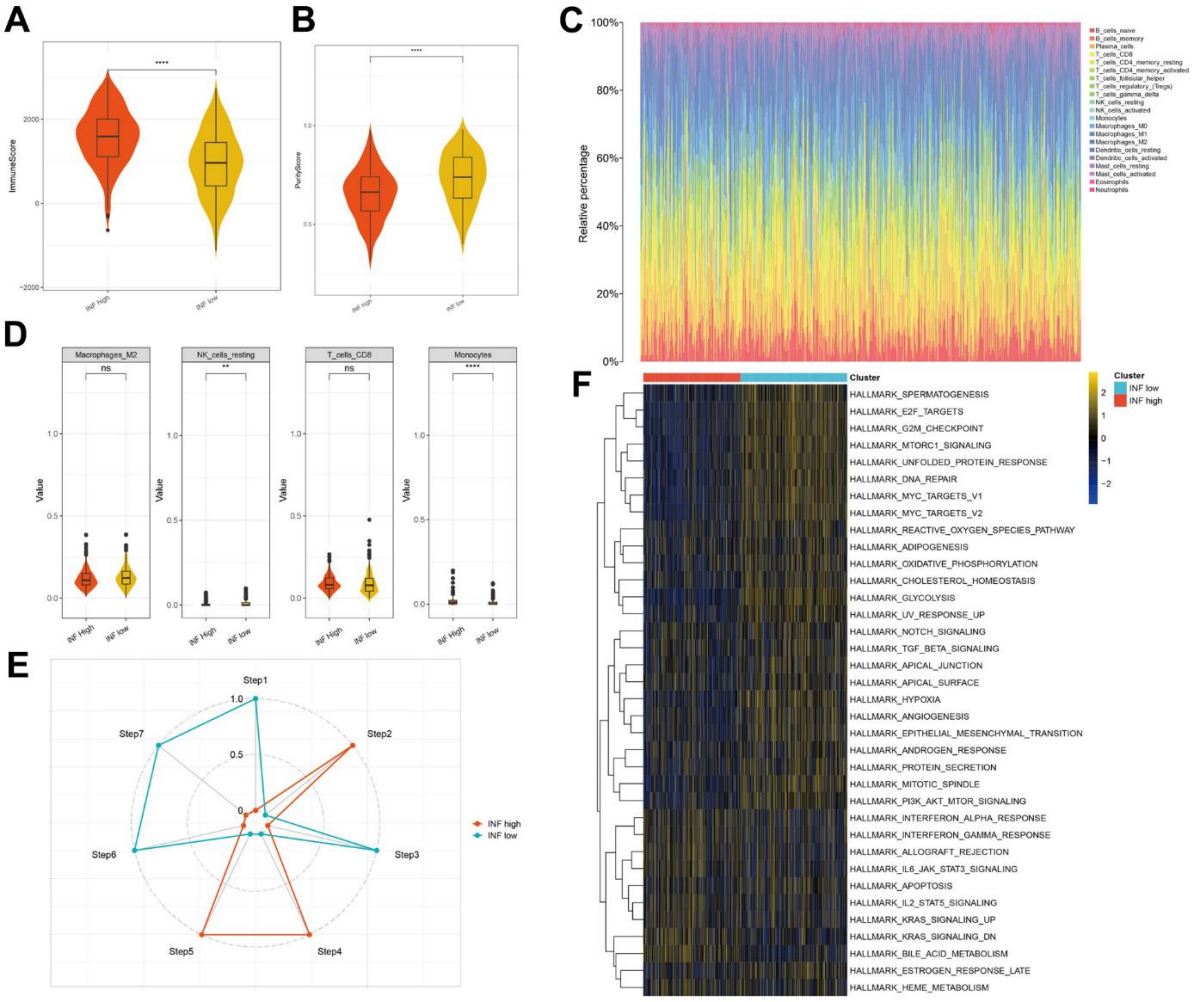
**The distinct TME features and anticancer immune activities of the two subtypes.** (**A**, **B**) Violin plots showing the immune score and tumor purity score of each subtype. (**C**, **D**) Immune infiltration in TCGA-LUAD samples. (**E**) Anticancer immune activity of the seven-step cancer-immunity cycle. (**F**) Heatmap of the 36 hallmark pathways differentially enriched between different inflammation subtypes identified by GSVA.

Inflammation markedly influences the TME, particularly the immune cells implicated in tumor evolution and progression. To elucidate the distinctions and relationships between the two subtypes, we analyzed their respective TME composition. The immune score notably declined from the INF-high to INF-low subtypes (Figure 3A), whereas tumor purity significantly increased (Figure 3B), suggesting higher immune cell infiltration in the INF-high subtype. We employed the CIBERSORT method to assess immune heterogeneity among the subtypes, which revealed variations in the infiltration of 22 immune cell types (Figure 3C). Specifically, the INF-low subtype showed elevated levels of immunosuppressive cells, such as M2-type macrophages, and resting immune cells, such as resting NK cells, indicating a potential immunosuppressive microenvironment driven by this TME composition (Figure 3D).

Subsequently, we assessed anticancer immune functions in the seven-step cancer immunity cycle for both subtypes using TIP. The INF-high subtype demonstrated elevated activity in steps 2 (presentation of tumor antigen), 4 (T cell transfer to tumors), and 5 (immune cell infiltration into tumors). In contrast, the INF-low subtype exhibited enhanced activity in steps 1 (antigen release from tumors), 3 (priming and activation), 6 (tumor cell detection by T cells), and 7 (tumor cell apoptosis) (Figure 3E). These observations suggest that mitigating the immunosuppressive microenvironment and boosting immune cell infiltration in the INF-low subtype may improve clinical outcomes in LUAD.

Additionally, we explored the pathways associated with the inflammation-based subtypes. The results of GSVA indicated that the INF-low subtype was significantly enriched for pathways negatively regulating immune responses, such as TGF-β signaling, hypoxia, epithelial-mesenchymal transition, and angiogenesis (Figure 3F).

### Identification of hub genes associated with inflammation in LUAD

**Figure 4:**
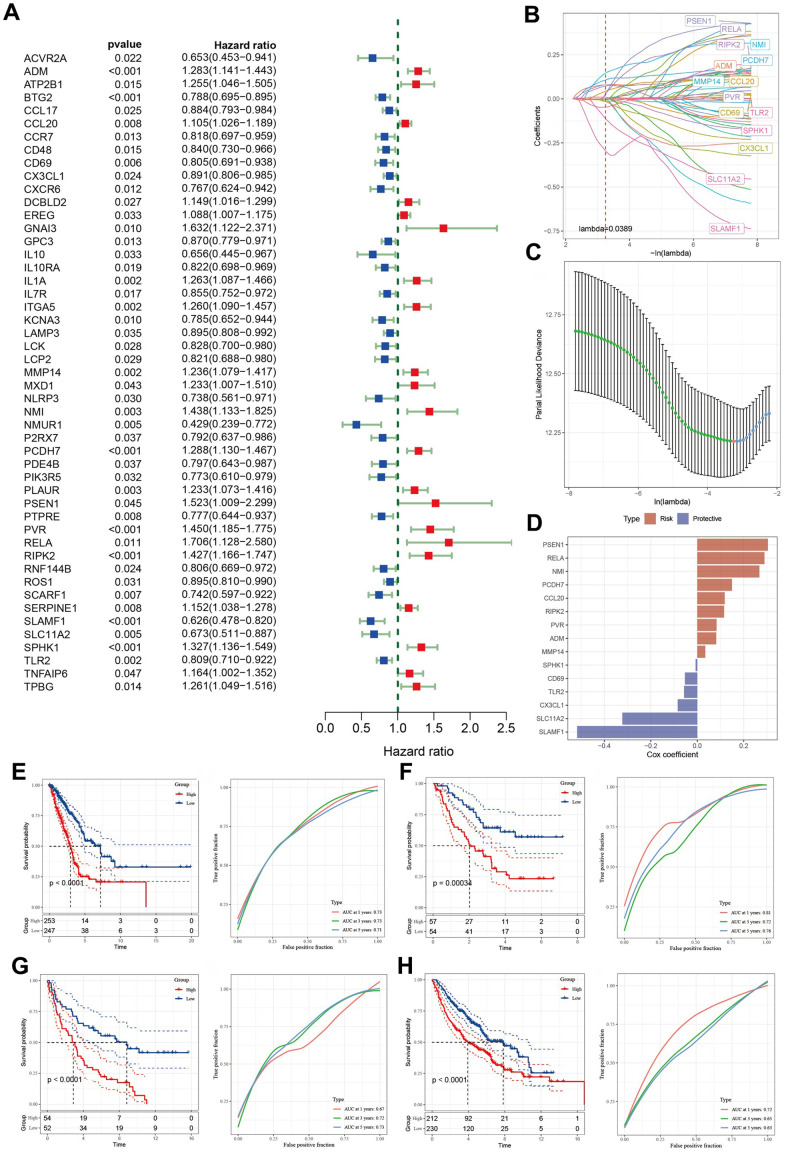
**Identification of the predictive hub genes for the construction of a risk signature.** (**A**) Forrest plot of prognosis-related inflammation genes identified by univariate Cox regression analysis. (**B**) The trajectory of each independent variable with lambda. (**C**) Plot of the generated coefficient distributions for the logarithmic (lambda) series for parameter selection. (**D**) Multivariate Cox coefficients for each gene in the risk signature. (**E**–**H**) Kaplan–Meier and ROC curves of the risk model constructed using the data of 15 genes from TCGA, GSE3141, GSE37745, and GSE68465 cohorts.

A total of 49 inflammation-related genes that significantly correlated with LUAD prognosis were initially identified using univariate Cox regression analysis (Figure 4A). Subsequently, LASSO Cox regression analysis was employed to refine this list, retaining 15 genes with a lambda value of 0.0389 (Figure 4B, 4C). The final 15-gene risk signature derived from multivariate Cox regression analysis using a stepwise regression method comprised ADM, CCL20, CD69, CX3CL1, MMP14, NMI, PCDH7, PSEN1, PVR, RELA, RIPK2, SLAMF1, SLC11A2, SPHK1, and TLR2 (Figure 4D). The risk score for each sample was calculated using the formula: Risk score = ADM*0.081382906 + CCL20*0.118144771 + CD69*(-0.05315822) + CX3CL1*(-0.084434545) + MMP14*0.0345135 + NMI*0.26713128 + PCDH7*0.149137053 + PSEN1*0.303754865 + PVR*0.082834768 + RELA*0.289113195 + RIPK2*0.115193023 + SLAMF1*(-0.516865874) + SLC11A2*(-0.322541796) + SPHK1*(-0.007444817) + TLR2*(-0.056765323). The samples were categorized into high- and low-risk groups following z-mean normalization. Kaplan–Meier survival analyses indicated that high-risk patients exhibited significantly worse survival outcomes than their low-risk counterparts in both TCGA and GEO cohorts (Figure 4E–4H). The model’s area under the curve (AUC) values for 1- to 5-year survival data ranged from 0.71 to 0.73, 0.72 to 0.81, 0.67 to 0.73, and 0.63 to 0.72 in TCGA, GSE3141, GSE37745, and GSE68465 cohorts, respectively (Figure 4E–4H).

### Responsiveness of risk signature to PD-L1 blockade immunotherapy

**Figure 5:**
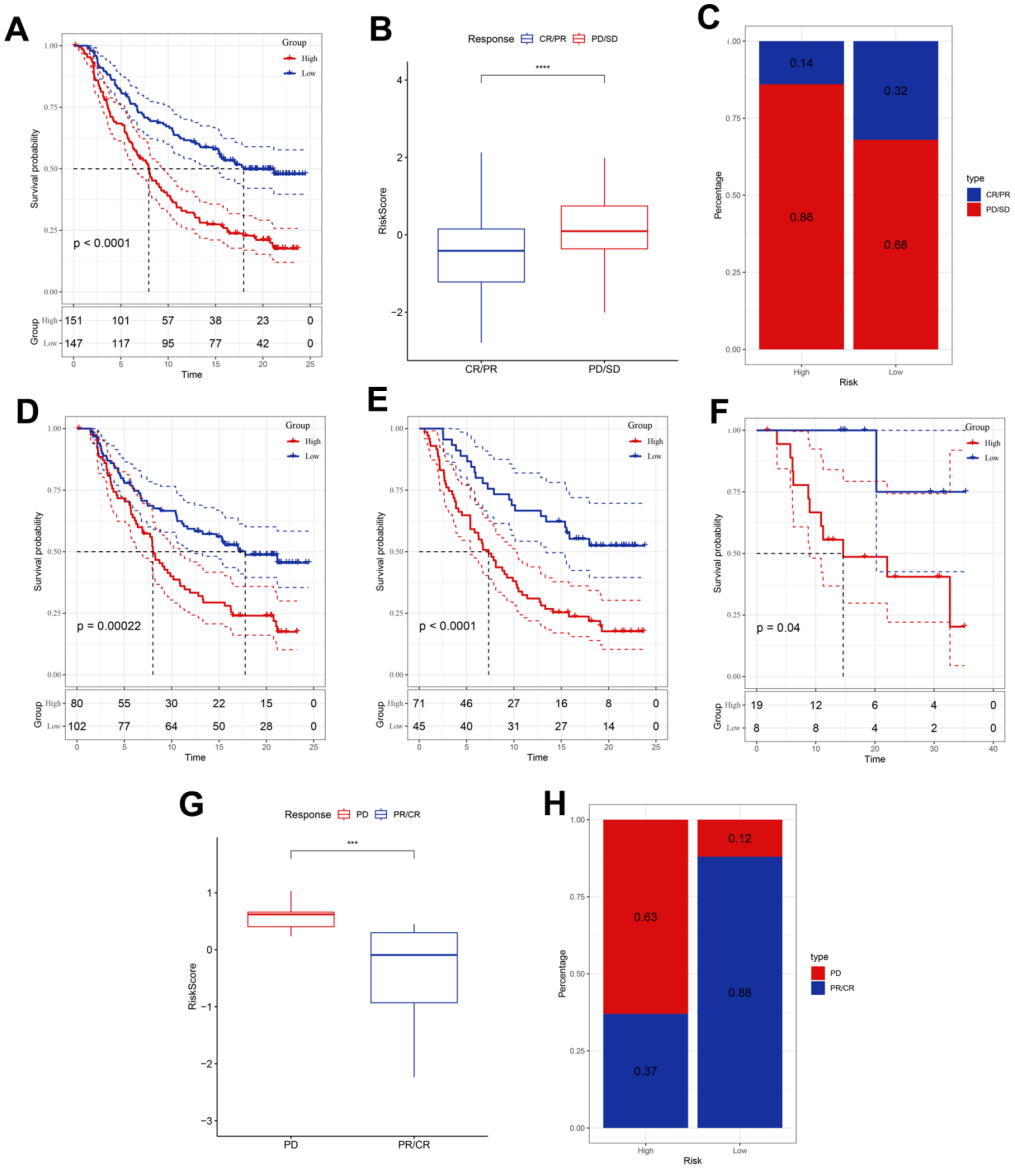
**Responsiveness of risk score to PD-L1 blockade immunotherapy in the IMvigor210 and GSE78220 cohorts.** (**A**) Prognostic difference among the risk score groups in the IMvigor210 cohort. (**B**) Differences in risk scores among immunotherapy responses in the IMvigor210 cohort. (**C**) Distribution of immunotherapy responses among the risk score groups in the IMvigor210 cohort. (**D**, **E**) Prognostic difference between the risk score groups in patients with early or advanced stage disease in the IMvigor210 cohort. (**F**) Prognostic difference among the risk score groups in the GSE78220 cohort. (**G**) Differences in risk scores among immunotherapy responses in the GSE78220 cohort. (**H**) Distribution of immunotherapy responses among the risk score groups in the GSE78220 cohort. ***P <0.001, ****P < 0.0001.

T-cell immunotherapy has gained prominence as an anticancer treatment that confers synergistic survival advantages. Therefore, we evaluated the prognostic relevance of our risk signature for immune checkpoint therapy by focusing on the IMvigor210 and GSE78220 cohort data. The 298 patients in the IMvigor210 cohort exhibited varied responses to anti-PD-L1 receptor blockers, including complete response (CR), partial response (PR), stable disease (SD), and progressive disease (PD). Analysis of the IMvigor210 cohort revealed that patients categorized into the low-risk group based on our risk model experienced substantial clinical benefits and markedly prolonged overall survival compared with that of their high-risk counterparts (Figure 5A). Patients with PD/SD had higher risk scores than those with CR/PR (Figure 5B). Furthermore, a greater proportion of PD/SD cases was observed in the high-risk group than in the low-risk group (Figure 5C). Notably, significant survival disparities were evident between the distinct risk groups for both stage I + II and stage III + IV patients (Figure 5D, 5E), suggesting that high-risk patients demonstrated suboptimal responses to anti-PD-L1 receptor blockers. This observation was true across both early and advanced stages of the disease. Similarly, in the GSE78220 cohort, the low-risk group exhibited significantly extended overall survival relative to that of the high-risk group (Figure 5F). Additionally, patients with PD displayed elevated risk scores compared to those shown by patients with PR/CR, and a higher incidence of PD was observed in the high-risk group than in the low-risk group (Figure 5G, 5H).

### Mutation, immunity, and pathway analysis of the 15 hub genes

**Figure 6:**
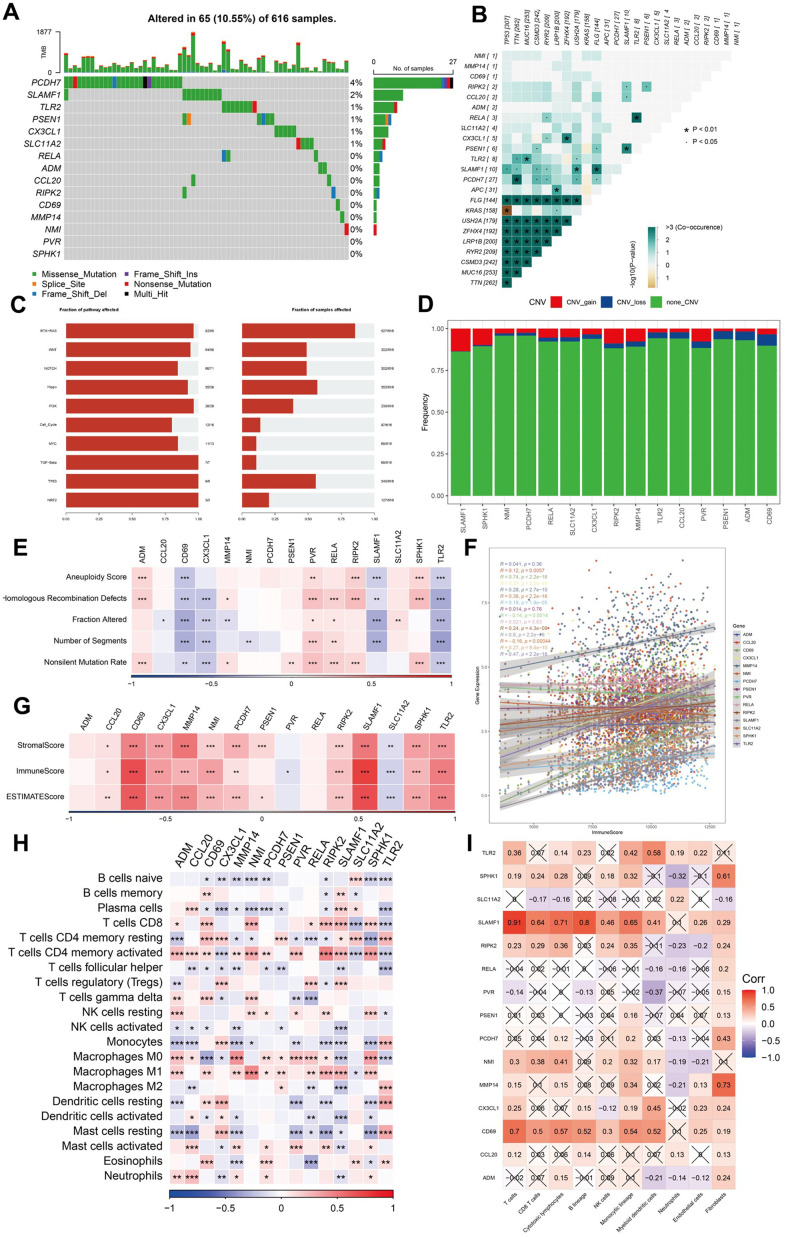
**Analysis of mutation, immunity, and hub gene pathways.** (**A**–**E**) The correlation between the mutation status and hub genes. (**F**–**I**) The features of immune cells and immune score based on the 15 hub genes. ***P <0.001, ****P < 0.0001.

We analyzed the single nucleotide variants (SNVs) of the 15 genes included in the risk signature and found that PCDH7 and SLAMF1 exhibited SNV mutations in multiple samples. Conversely, no SNV mutations were identified in PVR or SPHK1 (Figure 6A). Subsequently, we explored the co-occurrence probability between the 13 mutated genes and the 10 most frequently mutated genes. Notably, SLAMF1 displayed a high probability of co-occurrence with mutations in USH2A, FLG, TP53, TTN, CSMD3, and RYR2 (Figure 6B). We also assessed the mutation frequency in ten major oncogenic pathways and identified mutations in several pathways, including the RTK-RAS, Hippo, and TP53 pathways (Figure 6C). Among these 15 genes, SLAMF1 demonstrated the highest frequency of copy number variation (CNV) gain, whereas CD69 had the highest frequency of CNV loss (Figure 6D). We evaluated the correlation between these genes and multiple molecular signatures associated with LUAD. The correlation heatmap indicated that CD69 and TLR2 were significantly negatively correlated with aneuploidy score, homologous recombination defects, altered fraction, number of segments, and non-silent mutation rate. Conversely, PVR exhibited a significant positive correlation with these parameters (Figure 6E). The relationship between the immune score and expression levels of the 15 genes was also analyzed, revealing that most genes were positively correlated with the stromal, immune, and estimated scores. SLC11A2 and PVR expression negatively correlated with these scores (Figure 6F). We compared the immune scores between the different expression groups based on the median gene expression values. The high expression group of genes including C7, GPR34, SDS, and STOM demonstrated significantly elevated immune scores compared to those of the low expression group (Figure 6G). Additionally, SLAMF1 and RELA were significantly positively correlated with CD8+ T cells and M1 macrophages and negatively correlated with M2 macrophages (Figure 6H). The expression of SLAMF1 and CD69 positively correlated with the nine immune cell types (Figure 6I). Pathway analysis indicated significant correlations between these genes and 41 pathways, including the Notch and JAK-STAT signaling pathways (Supplementary Figure 1A, 1B).

### The 15-gene signature displayed a substantial correlation with immune-related characteristics

**Figure 7:**
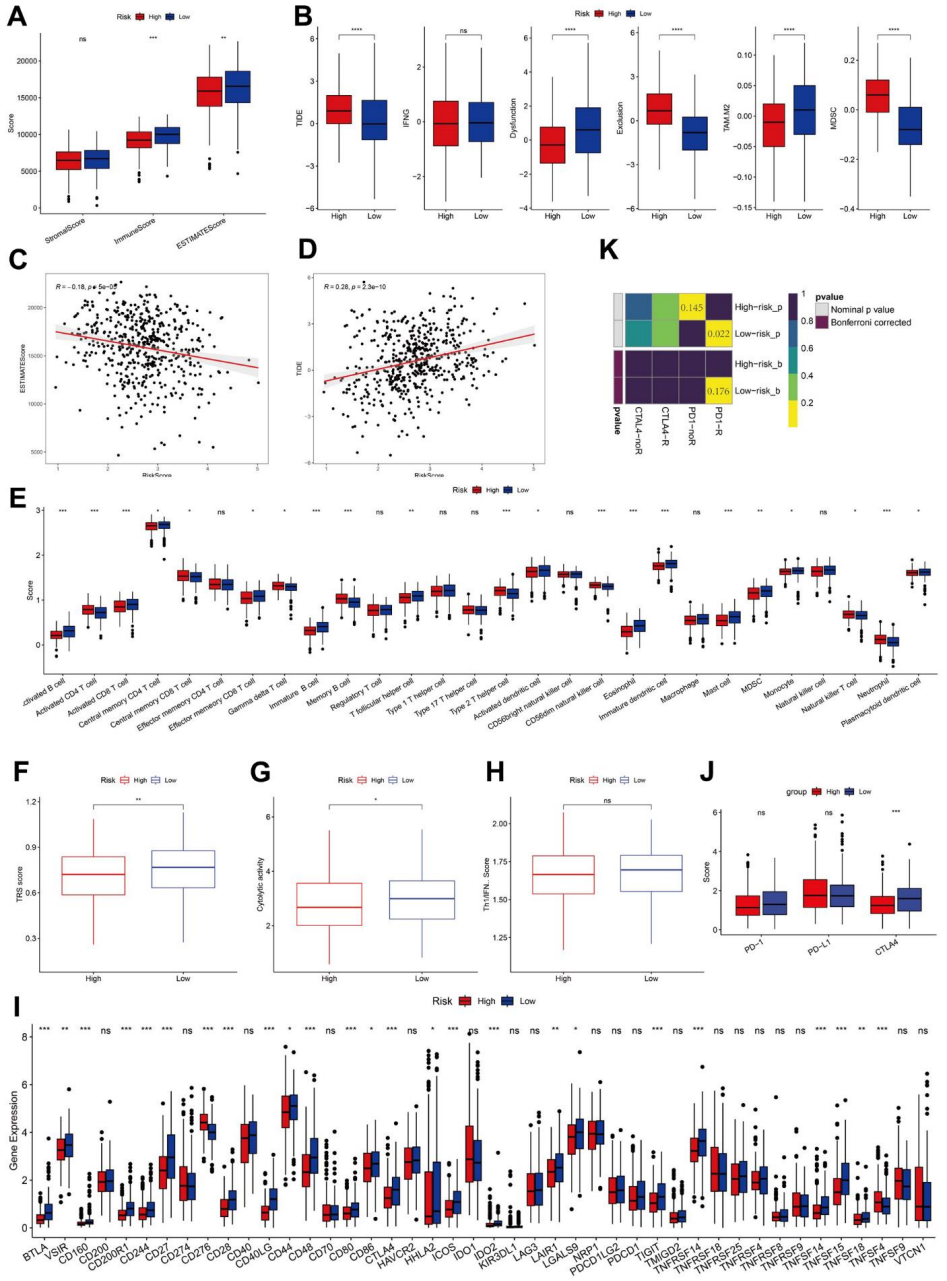
**Analysis of immunity status of risk groups.** (**A**–**D**) Immune status and immunotherapy between risk groups. (**E**–**H**) Active or suppressive TME between risk groups. (**I**, **J**) Exploration of immune checkpoints. (**K**) Submap analysis. *P <0.05, **P <0.01, ***P <0.001, ****P < 0.0001.

To assess the differences in immune status between the two risk groups, we used ESTIMATION and ssGSEA. Immune and stromal cells were evaluated, and their scores were combined to obtain the estimated score, which was higher in the low-risk group (Figure 7A). TIDE, a tool for predicting tumor patient sensitivity to immune checkpoint inhibitors (ICIs), showed that the response rate to immunotherapy was poorer in the high-risk group than in the low-risk group (Figure 7B) [26]. Further, the correlation between risk scores and the estimated and TIDE scores was significant (Figure 7C, 7D).

ssGSEA revealed that the low-risk group had a higher abundance of infiltrated immune cell types, such as activated CD8+T and B cells than that of the high-risk group (Figure 7E). Additionally, the low-risk group showed higher levels of TRS, CYT, and IFN-γ, indicative of a more immunoreactive microenvironment in the TCGA dataset (Figure 7F–7H) [20, 27]. The more immunoreactive TME may partially explain the better prognosis of patients in the low-risk group.

We analyzed the expression levels of immune checkpoints and found them to be elevated in the low-risk group, with CTLA4 being notably lower in the high-risk group (Figure 7I, 7J). Subclass mapping (submap) indicated a higher likelihood of response to ICIs in the low-risk group (Figure 7K).

GSVA showed that the high-risk group was enriched in pathways negatively modulating immune responses, such as TGF-β signaling and hypoxia, aligning with the INF-low subtype characteristics (Supplementary Figure 1C). The infiltration analysis results of immune and stromal cells in both risk groups performed using TIMER and MCP-counter are displayed in a heatmap. We found that patients in the low-risk group exhibited higher proportions of immune and stromal cell infiltration than those in the high-risk group (Supplementary Figure 1D). These findings suggest that the low-risk group, which belongs to the hot tumor subtype, may respond favorably to immunotherapy.

### Evaluating the sensitivity of chemotherapeutic drugs to patients with LUAD and finding potential drugs

To identify potential therapeutic agents for high-risk patients with LUAD, we analyzed sensitivity data sourced from the Cancer Therapeutics Response Portal (CTRP) and profiling of relative inhibition simultaneously in mixtures (PRISM) datasets. These datasets include sensitivity information for 481 and 1448 compounds across 835 and 482 cancer cell lines (CCLs), respectively [28]. We identified 5 agents from CTRP (BI-2536, KX2-391, leptomycin B, paclitaxel, and SB-743921) and 13 from PRISM (AT-9283, cabazitaxel, cyclocytidine, deforolimus, docetaxel, epothilone-b, gemcitabine, ispinesib, PF-03814735, PRT062070, R406, SNS-314, and vincristine) that showed a significant negative correlation with risk and lower estimated AUC values in the high-risk group than in the low-risk group (Supplementary Figure 2A–2D). To select suitable chemotherapeutic drugs for patients with LUAD, we assessed the half-maximal inhibitory concentration (IC50) of the four drugs in the two risk groups and explored the relationships between the 15 hub genes and these drugs (Supplementary Figure 2E). Our findings indicate that several genes influence sensitivity to chemotherapy. For instance, higher PVR expression correlated with resistance to multiple drugs such as tamoxifen and oxaliplatin but with increased sensitivity to irrofulvens. Similarly, elevated CD69 expression was linked to increased sensitivity to drugs such as nellarabine and dexamethasone decadron.

### Validation of inflammation-associated gene expression pattern by scRNA-seq analysis

**Figure 8:**
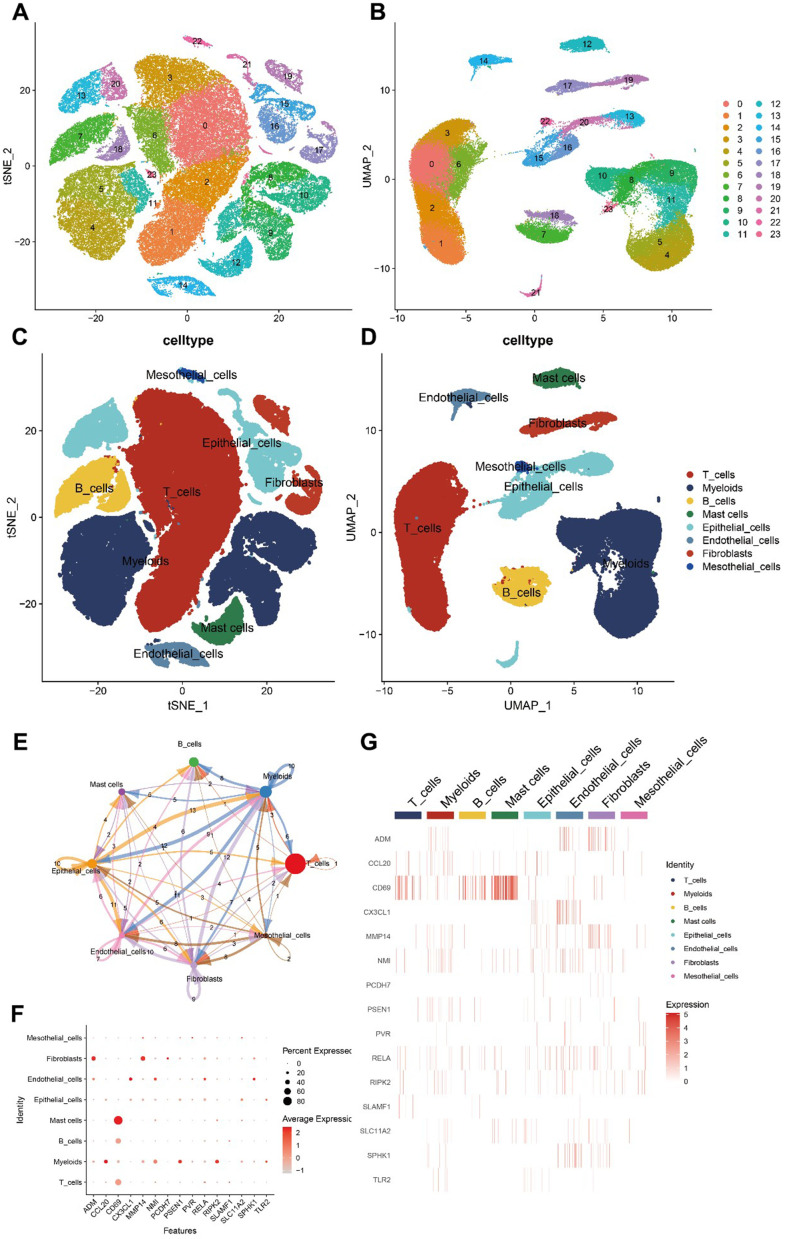
**Hub gene scRNA-seq analysis results.** (**A**–**D**) 24 clusters and eight cell types identified by tSNE and UMAP algorithms. (**E**) Analysis of cell communications. (**F**, **G**) Expression of hub genes in different cell types.

To validate the expression patterns of inflammation-related genes in distinct cell types within the TME, we analyzed the scRNA-seq data from patients with LUAD in the GSE131907 dataset. After quality control, data normalization, and PCA, cells from the LUAD samples were categorized into 24 clusters and 8 cell types using the tSNE and UMAP algorithms (Figure 8A–8D). We then assessed the intensity of cell communication among the eight cell types (Figure 8E) and determined the expression levels of 15 inflammation-related genes across these cell types. The analysis revealed that CD69 was significantly overexpressed in mast and T cells. Among the previously identified risk genes, PSEN1, CCL20, RIPK2, and NMI were mainly expressed in myeloid cells, whereas RELA was predominantly expressed in endothelial cells (Figure 8F, 8G).

### RELA was highly expressed in LUAD tissues and promotes the proliferation of tumor cells

Among the genes identified in our model, RELA, also known as transcription factor p65, is an essential effector molecule in the nuclear factor-kappa B (NF-kB) inflammatory signaling pathway [29]. NF-kB is a critical regulator of inflammation, cancer, and immunity, playing a pivotal role in various malignancies [30]. The most common form of NF-kB is a heterodimer consisting of the p50 and p65 subunits. Transcription factor p65 possesses transcriptionally active domains and is implicated in cell survival, invasion, proliferation, metastasis, angiogenesis, and chemoresistance. Therefore, we sought to explore the specific oncogenic effects of RELA in LUAD.

**Figure 9:**
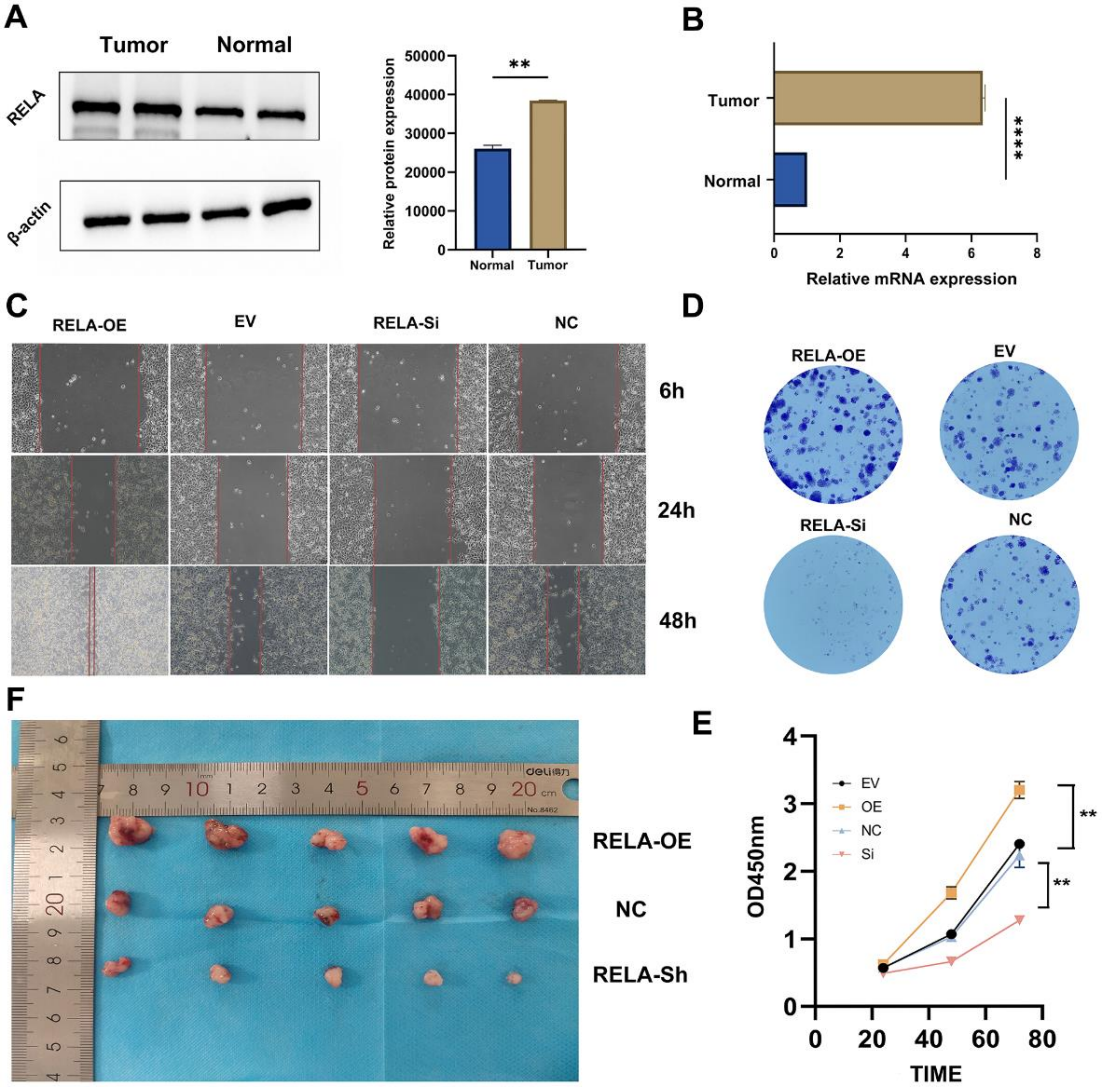
**Promotion of tumor cell proliferation by RELA.** (**A**, **B**) Differential expression of RELA in tumor vs. normal tissues, with pronounced upregulation in tumor samples. (**C**) Microscopic images highlighting cell migration differences across treatments over time. (**D**) Varying clonogenic potential among treatments, notably between RELA-OE and RELA-Si. (**E**) Results of the CCK-8 assay presenting enhanced proliferation in the RELA-OE group. (**F**) *In vivo* tumor size differences, with RELA-OE tumors being significantly larger.

Our initial findings indicated that both the RNA and protein encoded by RELA were highly expressed in LUAD tissues compared to those in normal tissues (Figure 9A, 9B). Functional assays, including CCK-8, wound healing, and colony formation assays, revealed that high RELA expression enhanced the proliferation and invasiveness of LUAD cells (Figure 9C–9E). To further validate the tumor-promoting effects of RELA, we subcutaneously injected mice with RELA-overexpressing or RELA-knockdown cells. Mice injected with RELA-overexpressing cells exhibited a larger average tumor volume and weight than those of the mice injected with RELA-knockdown cells (Figure 9F).

### RELA undergoes phase separation *in vitro* and *in vivo*, likely associated with its carcinogenic effect

A search of the UniProt website revealed that the protein encoded by RELA contains a significant number of intrinsically disordered regions (IDRs). As suggested by their names, these regions lack a defined three-dimensional (3D) structure and often have short amino acid motifs capable of mediating weak multivalent interactions. IDRs can facilitate protein interactions with other macromolecules, leading to liquid-liquid phase separation (LLPS) [31, 32]. We postulated that RELA could undergo phase separation and that this characteristic might be linked to its tumorigenic effects.

**Figure 10:**
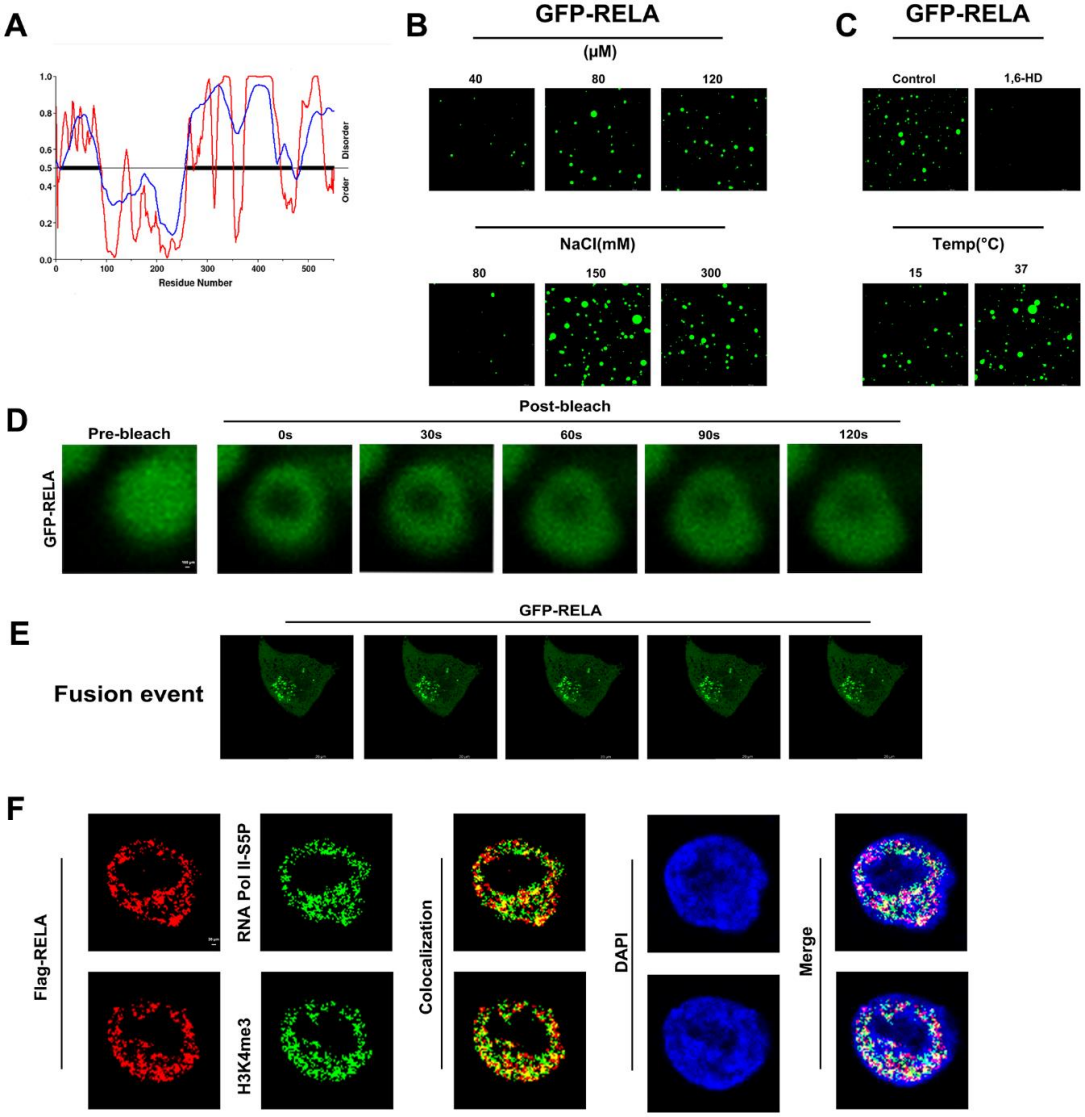
***In vitro* and *in vivo* phase separation results of RELA.** (**A**) Predicted phase separation ability of the RELA protein’s IDR region. (**B**, **C**) *In vitro* liquid-liquid phase separation (LLPS) of EGFP-tagged RELA under various physicochemical conditions. (**D**) *In vitro* fluorescence recovery after photobleaching (FRAP) of EGFP-RELA. (**E**) *In vivo* droplet fusion events of EGFP-RELA. (**F**) Co-localization of RELA with specific transcription elements, suggesting intricate relationships in gene expression regulation.

Analysis of the RELA primary sequence using IUPred and VSL2 indicated a significant propensity for structural disorder and high likelihood of phase separation (Figure 10A). Purified GFP-RELA spontaneously formed droplets in solution, with the size and number of droplets being dependent on the temperature and salt concentration. Importantly, the formation of these droplets was substantially inhibited by 5% 1,6-hexanediol, a compound known to disrupt weak hydrophobic interactions (Figure 10B, 10C). *In vitro* FRAP experiments confirmed that the condensates formed by the RELA exhibited liquid-like properties (Figure 10D).

To investigate whether phase separation ability is relevant to the biological function of RELA, we ectopically expressed GFP-RELA in H1299 cells and conducted immunofluorescence studies. Live-cell imaging showed that the RELA condensates readily fused into larger structures over time (Figure 10E). Additionally, the colocalization of RELA with the transcriptional activation markers H3K4me3 and RNA Pol II-S5P suggested that the condensates formed by RELA may recruit other transcription factors for activation (Figure 10F). In summary, our findings indicate that RELA can undergo phase separation to form droplets that activate transcription in the nucleus.

## DISCUSSION

LUAD continues to pose a formidable challenge in the field of oncology and is emerging as one of the most intricate and enigmatic malignancies. Owing to its multifaceted nature and complex etiology, LUAD requires a deeper exploration of its underlying mechanisms. Central to this quest is an understanding of the role of inflammatory responses, which have become increasingly recognized as pivotal contributors to patient prognosis and efficacy of therapeutic interventions. Our recent investigations have extensively delved into these aspects, providing novel and insightful insights into the molecular landscape of LUAD, particularly emphasizing the impact of inflammation.

Utilizing state-of-the-art consensus clustering techniques, we conducted a meticulous classification of LUAD into two prominent inflammation-based subtypes: INF-low and INF-high. This nuanced demarcation between the subtypes is underscored by contrasting inflammatory responses, which appear to have far-reaching implications for the clinical trajectory of LUAD. Through a rigorous analysis of our patient cohort, we observed that individuals categorized into these subtypes demonstrated strikingly divergent clinical outcomes, thereby underscoring the profound influence of inflammation on the pathology and progression of LUAD.

Exhaustive exploration of the TME of these subtypes has resulted in distinct and insightful features. The INF-low subtype was characterized by an immunosuppressive TME, consistent with the hypothesis that such a microenvironment can significantly compromise patient prognosis. This subtype was found to carry a high burden of oncogenic mutations, highlighting its potential for targeted therapeutic intervention. In contrast, the INF-high subtype exhibited robust antitumor immune activity, signifying its potential as a prime candidate for tailored immunotherapeutic approaches.

Among the 15 identified hub genes, we focused on the RELA gene to unravel LUAD complexity. Our findings revealed pronounced expression of RELA in LUAD tissues and its propensity to fuel tumor cell proliferation. In addition to expression level analysis, we investigated the unique ability of RELA to undergo phase separation both *in vitro* and *in vivo*, suggesting a molecular mechanism that could be intricately linked to its oncogenic potential.

Our investigation also drew attention to the pivotal correlation between the risk signature and efficacy of PD-L1 blockade immunotherapy. In the current era in which immunotherapies are increasingly becoming the frontline treatment for various cancers, understanding the molecular underpinnings of responsiveness is crucial. Our 15-gene signature revealed compelling associations with immune-centric characteristics, indicating its potential as a powerful tool for patient stratification and personalized therapy.

We also performed an exhaustive analysis of chemotherapeutic drug sensitivity in patients with LUAD. This aspect of our research holds promise for identifying novel therapeutic candidates, thereby expanding the repertoire of available treatment options. By exploring the sensitivities and resistances of LUAD cells to various chemotherapeutic agents, our study highlights potential pathways for intervention, thus providing indications for more effective and tailored therapeutic strategies.

In a quest for a granular understanding, our research utilized scRNA-seq analysis to validate the expression of inflammation-related genes. This approach offers detailed, cell-specific insights into the diverse and dynamic gene expression patterns in LUAD tumors. This level of granularity is pivotal in adding robustness and reliability to our findings and further emphasizing the complex interplay of inflammatory responses within the TME.

## Supplementary Material

Supplementary Figures
