# Supplementary material for: Inflammation-based lung adenocarcinoma molecular subtype identification and construction of an inflammation-related signature with bulk and single-cell RNA-seq data
Source: Aging (Albany NY). 2024 May 20;16(10):8822–42. doi: 10.18632/aging.205840 (PMC11164500; doi:10.18632/aging.205840)
Supplement: Supplementary Figures [file aging-16-205840-s001.pdf]

SUPPLEMENTARY FIGURES

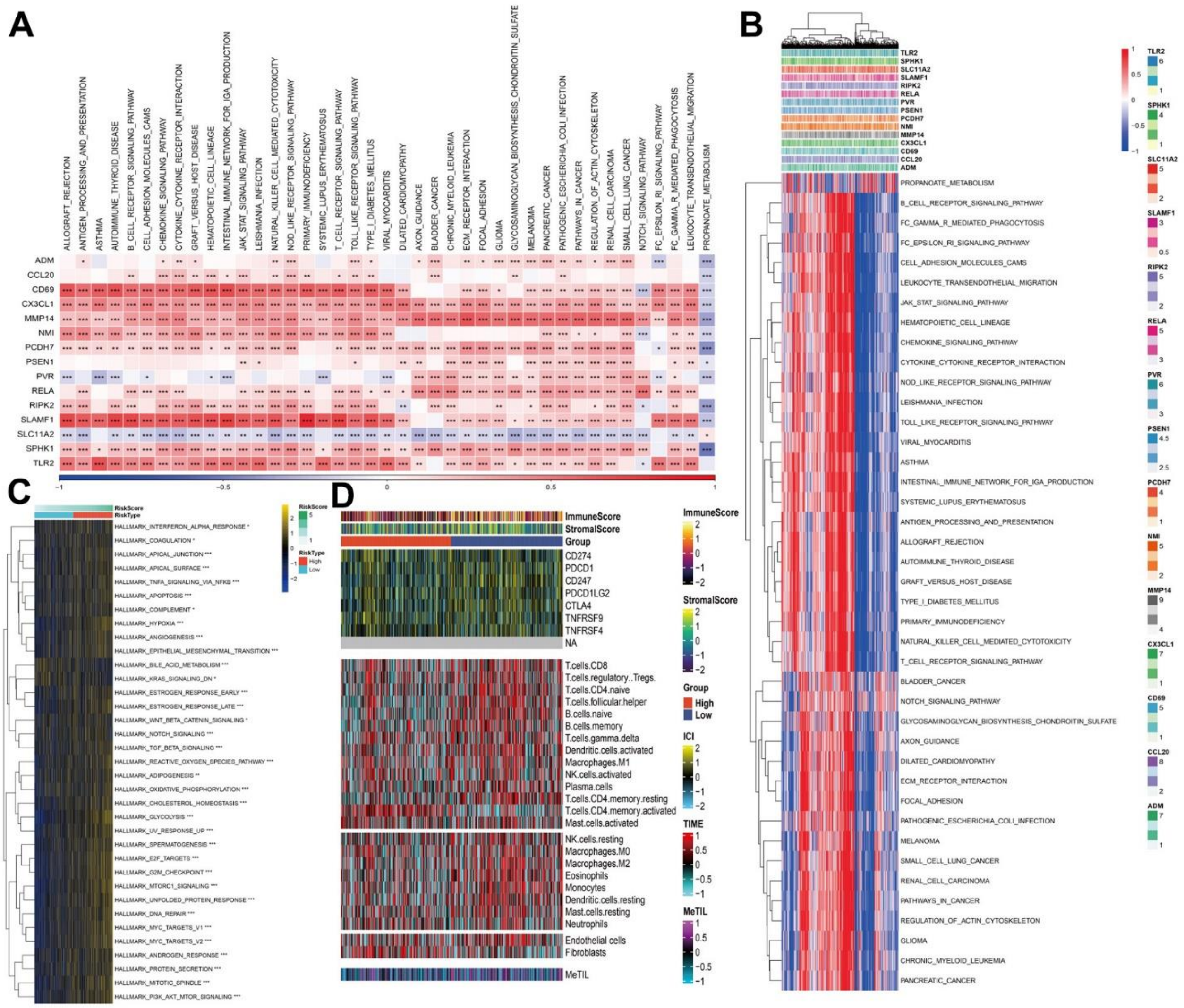

Supplementary Figure 1. Analysis of pathway enrichment and immune cell infiltration of the risk model. (**A**, **B**) The correlations between hub genes and 41 classical pathways. (**C**) The correlations between the two risk groups and hallmark pathways identified by GSVA. (**D**) Heatmap of immune cell expression in the TME in LUAD analyzed using multialgorithm, including existing data from TIMER and MCP-counter.

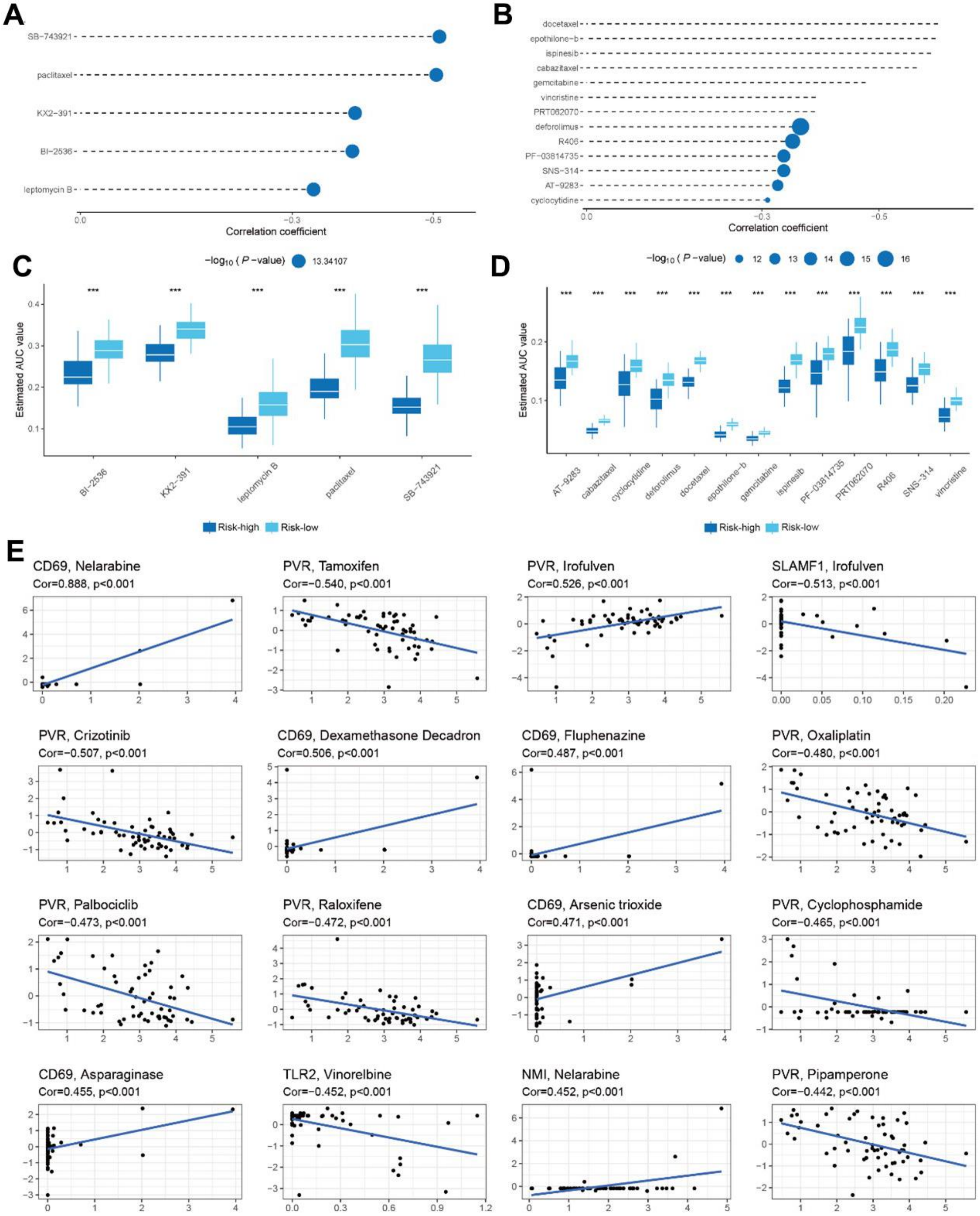

Supplementary Figure 2. Analysis of potential therapeutic agents. (**A**, **B**) CTRP- and PRISM-derived compounds. (**C**, **D**) Results of differential drug response analysis of CTRP- and PRISM-derived compounds; the lower values on the y-axis of boxplots imply greater drug sensitivity. (**E**) The correlation between hub genes and sensitivity to chemotherapy.
